# PCR combined with serologic testing improves the yield and efficiency of SARS-CoV-2 infection hunting: A study in 40,689 consecutive overseas arrivals

**DOI:** 10.3389/fpubh.2023.1077075

**Published:** 2023-02-13

**Authors:** Li-Li Fang, Jian-Hui Zhu, Min-Jing Cai, Jing-Wen Zhang, Long-Can Jiang, Zhang Dai, Yu Lin, Xian-Ming Liang

**Affiliations:** ^1^Department of Clinical Laboratory, The First Affiliated Hospital of Xiamen University, School of Medicine, Xiamen University, Xiamen, China; ^2^Xiamen Key Laboratory of Genetic Testing, School of Medicine, Xiamen University, Xiamen, China; ^3^Centre of Clinical Laboratory, Zhongshan Hospital of Xiamen University, School of Medicine, Xiamen University, Xiamen, China; ^4^Institute of Infectious Disease, School of Medicine, Xiamen University, Xiamen, China

**Keywords:** SARS-CoV-2, serologic test, PCR, screening algorithm, yield, efficiency

## Abstract

**Background:**

The global epidemiological situation of COVID-19 remains serious. The rapid hunting of SARS-CoV-2 infection is the key means for preventing transmission.

**Methods:**

A total of 40,689 consecutive overseas arrivals were screened for SARS-CoV-2 infection based on PCR and serologic testing. The yield and efficiency of different screening algorithms were evaluated.

**Result:**

Among the 40,689 consecutive overseas arrivals, 56 (0.14%) subjects were confirmed to have SARS-CoV-2 infection. The asymptomatic rate was 76.8%. When the algorithm based on PCR alone was used, the identification yield of a single round of PCR (PCR1) was only 39.3% (95% CI: 26.1–52.5%). It took at least four rounds of PCR to achieve a yield of 92.9% (95% CI: 85.9–99.8%). Fortunately, an algorithm based on a single round of PCR combined with a single round of serologic testing (PCR1+ Ab1) greatly improved the screening yield to 98.2% (95% CI: 94.6–100.0%) and required 42,299 PCR and 40,689 serologic tests that cost 6,052,855 yuan. By achieving a similar yield, the cost of PCR1+ Ab1 was 39.2% of that of four rounds of PCR. For hunting one case in PCR1+ Ab1, 769 PCR and 740 serologic tests were required, costing 110,052 yuan, which was 63.0% of that of the PCR1 algorithm.

**Conclusion:**

Comparing an algorithm based on PCR alone, PCR combined with a serologic testing algorithm greatly improved the yield and efficiency of the identification of SARS-CoV-2 infection.

## Introduction

Severe acute respiratory syndrome coronavirus 2 (SARS-CoV-2) is the causative agent of coronavirus disease 2019 (COVID-19), and its rapid transmission and high virulence have resulted in the ongoing COVID-19 pandemic (1). The global epidemiological situation of COVID-19 remains serious. The rapid discovery of SARS-CoV-2 infection and the quick isolation of patients and tracing of their close contacts are currently the most effective means for preventing transmission. In low-prevalence areas in particular, the identification of SARS-CoV-2 infection is crucial. Currently, algorithms based on PCR alone are widely used to diagnose COVID-19 and for the detection and quantification of SARS-CoV-2 RNA (2, 3). There are several limitations to the use of PCR alone for diagnosing SARS-CoV-2 infection, including the potential for false-negative results, which may be linked to inadequate nasopharyngeal sampling, varying levels of the virus at different anatomical sites and different times during the disease course, and the inability to diagnose pre- or asymptomatic infections (4–6). Therefore, the yield of PCR is unsatisfactory. Due to rapid transmission and strong infectiousness of the disease, failure to detect a SARS-CoV-2 infection could greatly decrease the efficacy of prevention. To achieve higher yields, PCR must be performed repeatedly in all subjects and should consume considerable human and material resources during the initial COVID-19 pandemic. Compared with PCR, serologic testing is relatively easier to perform and faster (7). Unlike PCR, which can detect only acutely infected persons, serologic tests help determine whether the individual being tested was previously infected, even if that person never showed symptoms (8). However, the potential for false-positive results in serologic tests limits their use in low-prevalence populations (9, 10). Both PCR- and serology-based methods have obvious defects, but they possibly complement each other throughout the disease course. The efficiency of screening algorithms based on PCR combined with serologic testing for identifying SARS-CoV-2 infection in practice is unclear. In our region, the government used PCR combined with a serologic testing algorithm to hunt SARS-CoV-2 infection in consecutive overseas arrivals between July 2020 and September 2020. Therefore, we investigated different screening algorithms from an economic perspective to evaluate whether PCR combined with a serologic testing algorithm could improve the yield and efficiency of the identification of SARS-CoV-2 infection.

## Methods

### Study design and participants

A total of 40,689 consecutive overseas arrivals, which were screened for SARS-CoV-2 infection based on PCR and serologic testing in Xiamen city between July 2020 and September 2020 were retrospectively investigated. All individuals underwent the first round of serologic testing, total antibodies against SARS-CoV-2 (Ab), and PCR tests to identify SARS-CoV-2 infection on the first day of entry. The individuals who were Ab-positive (Ab+) were assigned to a key screening population, followed up for 14 days, and subjected to multiple rounds of PCR that were serially determined at 3, 5, 7, 10, and 14 days after entry. The individuals who were Ab-negative (Ab-) underwent serologic testing at 7 days after entry and underwent PCR at 7 and 14 days after entry. All individuals were followed up for 28 days. When the PCR result was positive (PCR+), the individual was escorted directly to the hospital for a comprehensive evaluation and epidemiological investigation. Based on the COVID-19 Diagnosis and Treatment Plan (eighth edition) of the National Health Commission of the People's Republic of China, the subjects were diagnosed according to their epidemiological history, clinical symptoms, imaging findings, and laboratory test results. Individuals with SARS-CoV-2 infection included asymptomatic and symptomatic individuals. According to the neutralization test and epidemiological history, Ab-positive (Ab+) was classified as true-positive or false-positive. Previously infected individuals were defined as those with true-positive antibody results and positive neutralization test results, but no symptoms or signs of COVID-19 and negative RT-PCR results during the study period. Finally, 40,498 individuals were non-infected. The age of the subjects ranged from 1 to 93 years, with a median age of 29 years, and 17,473 (43.1%) individuals were women. For the previously infected, the ages ranged from 21 to 65 years, with a median age of 34 years, and 65 (44.4%) individuals were women. To investigate different screening algorithms from an economic perspective, the testing algorithm for screening SARS-CoV-2 infection was set to nine algorithms, according to round and combination of PCR and serologic testing (Table 1). To determine the incremental yield of each SARS-CoV-2 test algorithm, we determined the number of additional SARS-CoV-2 infections detected relative to the number detected with the single round of PCR (PCR1). To determine the efficiency of each algorithm, we determined the number of serology and PCR tests used and the number needed to test (NNT) to detect one case of SARS-CoV-2 infection for each test.

**Table 1:** The screening algorithm for SARS-CoV-2 infection.

**Algorithm**	**PCR**		**Serologic testing**	
	**Rounds**	**Days**	**Rounds**	**Days**
PCR1	1	1	0	
PCR2	2	1, 3	0	
PCR3	3	1, 3, 5	0	
PCR4	4	1, 3, 5, 7	0	
PCR5	5	1, 3, 5, 7, 10	0	
PCR6	6	1, 3, 5, 7, 10, 14	0	
Ab1 + PCR1^a^	1	1	1	1
Ab2 + PCR2^a^	2	1, 7	2	1,7
Ab2 + PCR3^a^	3	1, 7, 14	2	1,7

a The individuals who were Ab-positive (Ab+) were assigned to a key screening population, followed up for 14 days, and subjected to multiple rounds of PCR. All were followed up for 28 days.

### PCR assays for SARS-CoV-2

Upper respiratory tract (URT) samples were collected from both nasopharyngeal and oropharyngeal swabs collected by trained medical staff (physicians and nurses). For lower respiratory tract (LRT) specimens, participants were given instructions the night before to collect the first morning sputum samples (after gargling) in a specimen cup. The Stream SP96 automatic nucleic acid extraction instrument (Da An Gene Co., Ltd. Guangzhou, China) was used for nucleic acid extraction. RT-qPCR was conducted using the SLAN-96P real-time PCR system (Shanghai Hongshi Biotechnology Co., Ltd., Shanghai, China). PCR assays for SARS-CoV-2 were performed using the Liferiver 2019-nCoV assay (Shanghai ZJ Bio-Tech Co., Ltd, Shanghai, China) to determine the presence of the virus through the identification of three genetic markers: the envelope (env) gene, the open reading frame (ORF) 1ab gene, and the nucleocapsid protein (N) gene. The cycle threshold (Ct) determined during RT-PCR testing refers to the cycle in which the detection of viral amplicons occurs, and it is inversely correlated with the amount of RNA present. A lower Ct value indicates larger quantities of viral RNA. The results were considered positive when the Ct values of all genes were <40 cycles. The assay had a sensitivity of 89.3% and a specificity of 100.0%, and no cross-reactivity was observed in clinical diagnostic efficacy (11). Two consecutive single-site positives or double-site positives are judged to indicate RT-PCR positivity according to the COVID-19 Prevention and Control Plan (eighth edition).

### Serologic testing

Blood samples were centrifuged at 3,000 rpm, and the upper serum layer was used for testing. The total antibodies against SARS-CoV-2 were measured using a Wantai^®^ Caris 200 system, based on a chemiluminescence microparticle immunoassay (CMIA) (Wantai Biological Pharmacy Enterprise Co., Ltd, Beijing, China). The detection experiments were performed according to the manufacturer's instructions. The kit was provided by Wantai Biological Pharmacy Enterprise Co., Ltd, Beijing, China. TAb detection was based on a double-antigen sandwich immunoassay using two types of mammalian cell-expressed recombinant antigens containing the receptor-binding domain (RBD) of the spike protein of SARS-CoV-2 as the immobilized and HRP-conjugated antigens. The antibody titer was calculated according to the cutoff and was recorded as the cutoff index (COI). COI<1.00 was considered negative, and COI≥1.00 was considered positive. The assay had a sensitivity of 96.7% and a specificity of 99.5%, and no cross-reactivity was observed (12).

### Statistical analysis

Statistical analysis was carried out using IBM SPSS Statistics version 20 (SPSS, Inc., Chicago, IL, USA) and GraphPad Prism version 8.00 (GraphPad Software, San Diego, CA, USA). Continuous variables that did not follow a normal distribution are reported as medians with interquartile ranges (IQRs). The statistical analysis for group comparisons was conducted using the Kruskal–Wallis and Mann–Whitney U-tests. To determine the diagnostic yields of different algorithms, the clinical diagnostic results were used as the gold standard according to their epidemiological history, clinical symptoms, imaging findings, and laboratory test results. We compared the differences in the proportions of infection detected by each algorithm relative to the single round of PCR (PCR1) algorithm using the McNemar chi-square test of paired proportions. Statistical significance was indicated by a *p*-value of <0.05 (<0.05).

## Results

### Outcomes of SARS-CoV-2 screening

**Figure 1:**
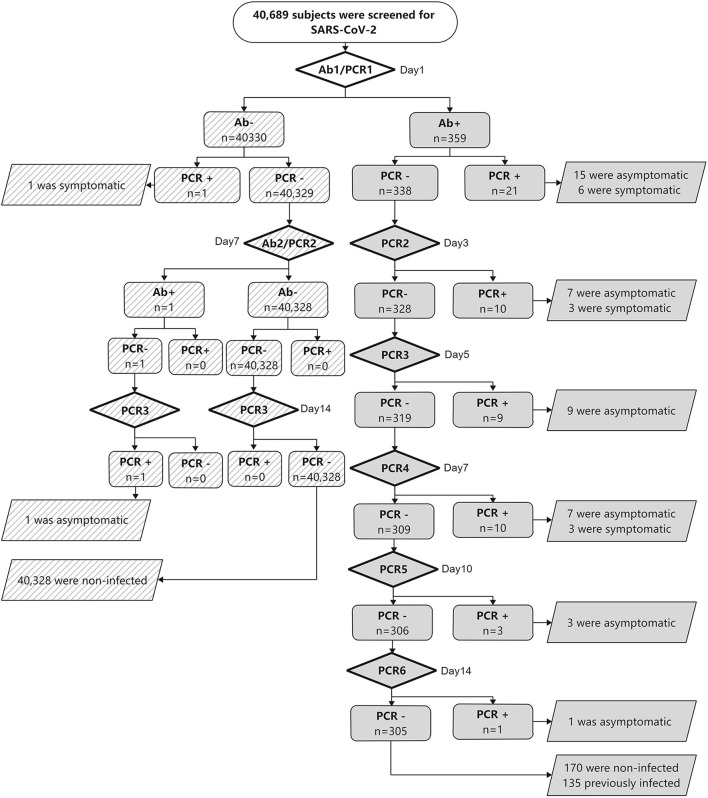
Results of SARS-CoV-2 screening. Abbreviations: SARS-CoV-2, severe acute respiratory syndrome coronavirus 2; PCR, polymerase chain reaction to detect SARS-CoV-2; Ab, total anti-SARS-CoV-2 antibody level.

We performed PCR and serologic tests on samples collected from 40,689 subjects between July 2020 and September 2020. A total of 56 (0.14%) subjects with SARS-CoV-2 infection were identified. They came from eight foreign countries (Table 2). The asymptomatic rate was 76.8%. There were no new cases of SARS-CoV-2 infection found among the remaining subjects during the 28-day follow-up (Figure 1). The first round of serology tests revealed 359 subjects to be Ab-positive. Among Ab-positive subjects, 54 (96.4%) subjects with SARS-CoV-2 infections were identified (Figure 1). Notably, 14 (25.0%) subjects with SARS-CoV-2 infections were discovered after more than three rounds of PCR were performed because Ab positivity was classified as the key subject, which was screened by multiple rounds of PCR. In addition, four (7.14%) subjects with SARS-CoV-2 infections were found after four rounds of PCR (Figure 1). On the other hand, among the 40,330 Ab-negative subjects, two subjects showed seroconversion within a week, both of whom were confirmed SARS-CoV-2 infection, one was confirmed on the first round of PCR, and the other was confirmed on the third round of PCR.

**Table 2:** Characteristics of 56 SARS-CoV-2 infections.

**Characteristics**	**Asymptomatic**	**Symptomatic**
*N* (%)	43 (76.8%)	13 (23.2%)
Male/Female	32/11	8/5
Age median (range) (years)	26 (8-64)	36 (25-64)
**Citizenship**		
Chinese	39 (90.7%)	12 (92.3%)
Non-Chinese	4 (9.3%)	1 (7.7%)
**Origin**		
Philippine	18 (41.9%)	11 (84.6%)
Singapore	14 (32.6%)	0 (0.0%)
United States	3 (7.0%)	1 (7.7%)
Other countries^a^	8 (18.6%)	1 (7.7%)

a Other countries included Mexico, Serbia, Australia, Kazakhstan, and Bangladesh.

### The yield of the SARS-CoV-2 screening algorithm

**Figure 2:**
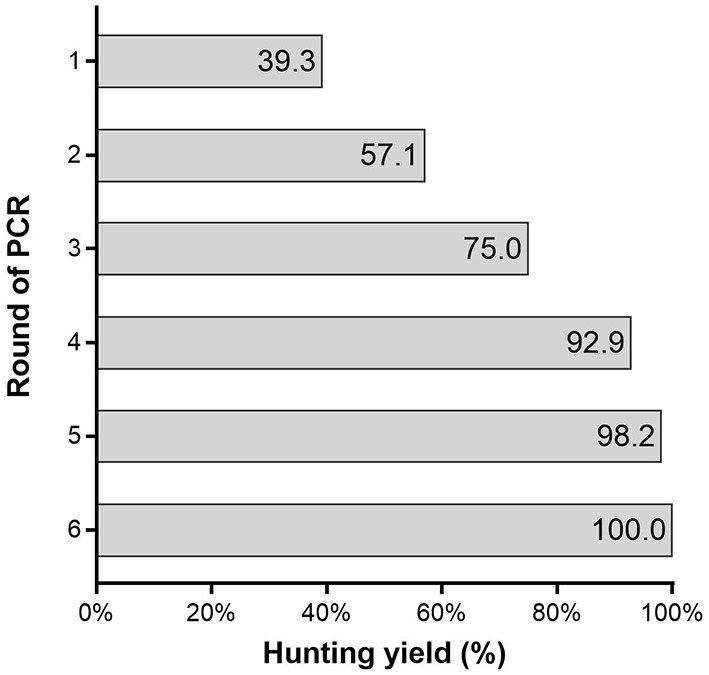
The round of PCR and hunting yield.

The identification yield of a single round of PCR was only 39.3% (95% CI: 26.1–52.5%). It took at least four rounds of PCR to achieve a yield of 92.9% (95% CI: 85.9–99.8%) (Figure 2). The diagnostic yield of the algorithm based on PCR alone increased as the number of PCR rounds increased. It was noted that the application of an algorithm based on PCR combined with serologic testing greatly improved the screening yield to 98.2% (95% CI: 94.6–100.0%). However, with the addition of serologic testing, the number of false positives increased to 305, which was 5.4 times greater than the number of SARS-CoV-2 infections (Table 3).

**Table 3:** Hunting yield, incremental yield, and number of false positives obtained with different algorithms.

**Strategy**	**Hunting yield**		**Incremental yield**			**Total false positives (n)**
	***n***	**% (95% CI)**	**Additional cases hunted (n)**	**Additional cases hunted (%) (95% CI)**	***p*** **-value of the difference**	
PCR1	22	39.3 (26.1–52.5)	REF^a^	REF		0
PCR2	32	57.1 (43.8–70.5)	+10	+17.9 (7.5–28.2)	0.00%	0
PCR3	42	75.0 (63.3–86.7)	+20	+35.7 (22.8–48.7)	<0.0001	0
PCR4	52	92.9 (85.9–99.8)	+30	+53.6 (40.1–67.0)	<0.0001	0
PCR5	55	98.2 (94.6–100.0)	+33	+58.9 (45.6–72.2)	<0.0001	0
PCR6	56	100.0 (100.0–100.0)	+34	+60.7 (47.5–73.9)	<0.0001	0
Ab1+PCR	55	98.2 (94.6–100.0)	+33	+58.9 (45.6–72.2)	<0.0001	305
Ab2+PCR2	56	100.0 (100.0–100.0)	+34	+60.7 (47.5–73.9)	<0.0001	305
Ab2+PCR3	56	100.0 (100.0–100.0)	+34	+60.7 (47.5–73.9)	<0.0001	305

a Results were expressed relative to the algorithm of a single round of PCR (PCR1) that was set REF.

n, number; SARS-CoV-2, severe acute respiratory syndrome coronavirus 2; PCR, polymerase chain reaction for SARS-CoV-2 detection; Ab, total anti-SARS-CoV-2 antibody level.

### The cost of different SARS-CoV-2 test algorithms

For the algorithm based on a single round of PCR, 40,689 tests were conducted at a cost of 3,865,455 yuan, and 1,850 tests costing 175,703 yuan were required to detect each case. However, this approach missed 34 (60.7%) SARS-CoV-2 infection cases. To achieve higher yields, the number of tests and the cost associated with the algorithm based on PCR alone rapidly increased as the number of rounds of PCR increased (Table 4). In four rounds of PCR (PCR4) achieving a yield of 92.9%, 162,660 tests were conducted at a cost of 15,452,700 yuan, and 3,128 tests costing 297,167 yuan were requiredfor hunting each case. Relative to the PCR1 algorithm, the test number and cost were increased 4.00-fold and 1.69-fold, respectively. Fortunately, the algorithm based on serologic testing combined with PCR was more effective. With a 98.2% yield, the algorithm based on a single round of PCR combined with a single round of serologic testing (PCR1+ Ab1) required 42,299 PCR and 40,689 serologic tests that cost 6,052,855 yuan. With a similar yield, the cost of PCR1+ Ab1 was 39.2% of that of four rounds of PCR. For hunting one case, 769 PCR and 740 serologic tests were required and cost 110,052 yuan, which was 63.0% of that of the PCR1 algorithm. For the algorithm based on PCR combined with serologic testing, the number of tests required and the cost of discovering one case increased as the round of testing increased, but the yield did not increase significantly (Table 4).

**Table 4:** The costs of SARS-CoV-2 testing algorithms.

**Strategy**	**Number of tests used**		**NNT of hunting one case**		**Test cost** ^**a**^		**Cost for hunting one case^**a**^**	
	**PCR**	**Ab**	**PCR**	**Ab**	**Total (yuan)**	**Relative** ^b^	**Total (yuan)**	**Relative** ^b^
PCR1	40,689	0	1,850	0	3,865,455	1.00	175,703	1.00
PCR2	81,356	0	2,542	0	7,728,820	2.00	241,526	1.37
PCR3	122,013	0	2,905	0	11,591,235	3.00	275,982	1.57
PCR4	162,660	0	3,128	0	15,452,700	4.00	297,167	1.69
PCR5	203,297	0	3,696	0	19,313,215	5.00	351,149	2.00
PCR6	243,931	0	4,356	0	23,173,445	6.00	413,812	2.36
PCR1 + Ab1	42,299	40,689	769	740	6,052,855	1.57	110,052	0.63
PCR2 + Ab2	81,356	81,018	1,453	1,447	11,779,720	3.05	210,352	1.20
PCR3 + Ab2	122,013	81,018	2,179	1,447	15,642,135	4.05	279,324	1.59

a Assume 95 yuan per PCR test and 50 yuan per Ab test, according to the standard government charge.

b Results were expressed relative to the algorithm of a single round of PCR (PCR1) that was set to 1.00.

N, number; NNT, number needed to test; SARS-CoV-2, severe acute respiratory syndrome coronavirus 2; PCR, polymerase chain reaction for SARS-CoV-2 detection; Ab, total anti-SARS-CoV-2 antibody level.

### Serologic testing in the population

**Figure 3:**
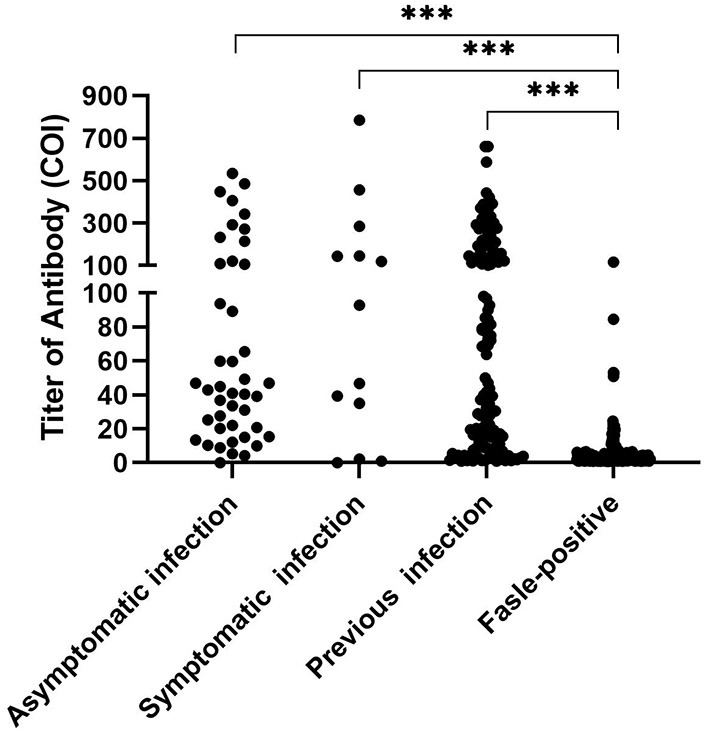
The titer of anti-SARS-CoV-2 antibody. Statistically significant differences correspond to the differences in the antibody titer between the false-positive group and the other groups. Abbreviations: COI, cut-off index. ***p < 0.001.

A total of 170 subjects showed false-positive results, and the false-positive rate was 0.42%. The Ab titers of the asymptomatic, symptomatic, previously infected, and false-positive groups were 43.00 (20.34–109.00) COI, 92.92 (18.63–215.20) COI, 49.98 (10.37–156.8) COI, and 1.99 (1.25–4.28) COI, respectively. A significantly lower titer was found in the false-positive group than in the other groups (Figure 3), with 94.70% of the subjects in the false-positive group showing a value below 20 COI. Within 1 week, seroconversion occurred in one case in the symptomatic group and one case in the asymptomatic group, and the positive rate of SARS-CoV-2 infection reached 100% (Table 5).

**Table 5:** Serology testing results of the population.

**Group**	**Ab1**		**Ab2**	
	**N of pos**	**Pos rate (%)**	**N of pos**	**Pos rate (%)**
Asymptomatic (*n* = 43)	42	97.7	43	100.0
Symptomatic (*n* = 13)	12	92.3	13	100.0
Previously infected (*n* = 135)	135	100.0	135	100.0
Non-infected (*n* = 40,498)	170	0.4	170	0.4

Ab, anti-SARS-CoV-2 antibody; N, number; Pos, positive; Ab1, the first antibody result after 1 week of isolation; Ab2, the second antibody result 1 week after the first antibody test.

## Discussion

The rapid hunting of SARS-CoV-2 infection and the quick isolation of patients are currently the most effective means for preventing the transmission of COVID-19 (13, 14). Different countries and regions adopted different SARS-CoV-2 screening algorithms and had different effects (15, 16). The successful hunting of SARS-CoV-2 infection depends heavily on the use of accurate tests performed at the appropriate time. In our study, the diagnostic yield of the algorithm based on one round of PCR was only 39.3%, indicating poor efficiency. At least four rounds of PCR (PCR4) were required to achieve a yield of over 90.0%. The diagnostic yield of the algorithm based on PCR alone increased as the number of PCR cycles increased. Furthermore, relative to the PCR1 algorithm, the test number and cost were increased 4.00 and 1.69-fold, respectively. This is a huge burden for any institution or country. Thus, the feasibility of this approach is highly questionable. Furthermore, 7.14% of infected subjects were missed, representing a huge hidden danger in epidemic control. The highest sensitivity of PCR testing based on nasopharyngeal sampling is observed from 0 to 4 days post-symptom onset, at 89%, dropping to 54% after 10 to 14 days (17). In the later stage of SARS-CoV-2 infection, the sensitivity of PCR was low and might not facilitate the identification. It suggested that the algorithm based on PCR alone could not meet the screening requirements.

Fortunately, it was noted that the use of an algorithm based on serologic testing combined with PCR could easily improve the screening yield to over 90.0%. The diagnostic yield of the algorithm based on a single serologic test combined with a single round of PCR was 98.2%. The success of diagnosis depends heavily on test accuracy and the use of accurate tests at the appropriate time. Serological tests show the lowest sensitivity at 0–7 days after symptom onset and the highest at >14 days (2, 3, 18). The sensitivity of IgG-, IgM-, and TAb-based tests is 25, 34, and 36%, respectively, during the first 7 days after symptom onset but increases to 62, 65, and 80% at 8–14 days post-symptom onset and 90, 85, and 93% after 14 days post-symptom onset (7, 18). In the early stage of SARS-CoV-2 infection, the sensitivity of serological testing was low and might not facilitate the identification. Fortunately, the advantages and disadvantages of PCR and serologic tests complement each other, covering all stages of the disease. The other reasons for the improvement achieved by the combined strategy were as follows. First, the amount of anti-SARS-CoV-2 antibody produced by asymptomatic individuals is similar to that produced by symptomatic individuals. Second, a highly sensitive detection method was used in this procedure. Third, the test time was several days after travel from the site of origin to the destination.

Furthermore, the algorithm based on serologic testing combined with PCR was more effective. With a 98.2% yield, the algorithm based on a single round of PCR combined with a single round of serologic tests (PCR1+ Ab1) required 42,299 PCR and 40,689 serologic tests that cost 6,052,855 yuan. With a similar yield, the cost of PCR1+ Ab1 was 39.2% of that of four rounds of PCR. For hunting one case, 769 PCR and 740 serologic tests were required at a cost of 110,052 yuan, which was 63.0% of that of the PCR1 algorithm. It is worth mentioning that by adding serologic testing, the size of the focus population was reduced from 40,689 to 361. Relative to the algorithm based on PCR alone, the combined approach substantially decreased the number of PCR tests required to obtain a similar yield. Compared with PCR, serologic testing is easier to perform and faster. In addition, blood samples are stable, easy to store, and less likely to contain infectious SARS-CoV-2 virus than respiratory specimens, decreasing the potential risk of infection for laboratory staff (7). Thus, the use of an algorithm based on serologic testing combined with PCR is more practical and inexpensive in the initial epidemic.

With the addition of serologic testing, the number of false positives was increased to 305, which was 5.4-fold more than that of SARS-CoV-2 infections. Serology is another important method for the investigation of SARS-CoV-2 infections (19). Nevertheless, the value of SARS-CoV-2 antibody testing for diagnosis, prevention, and control remains controversial (20). In the present study, total anti-SARS-CoV-2 antibody levels were measured, and the sensitivity for asymptomatic and symptomatic patients was 97.7 and 92.3%, respectively, which is higher than those reported previously (18, 21, 22). However, 170 subjects showed false positives, corresponding to a false-positive rate of 0.42%. Considering the 0.14% prevalence identified in this study, false positives were a great confounding factor. This suggests that serologic testing is not recommended as the primary approach for the diagnosis of SARS-CoV-2 infection (23, 24). A low titer was one of the characteristics of false positivity in the study. If a risk assessment dictates an overriding concern, the cutoff value can be set accordingly (10, 25). To exclude false positives, the cutoff value should be evaluated according to the specific objective and population.

This study has some limitations. First, because the time of subjects was in the early stage of the COVID-19 pandemic, a vaccinated population was not included in this study, and the variants were not involved. Second, the subjects came from all over the world and were highly mobile; thus, it was not possible to obtain a detailed disease course. Third, before boarding their plane, some subjects had been screened for SARS-CoV-2, which caused the true prevalence to be underestimated. Finally, due to the low number of SARS-CoV-2 infections, the relationship between the diagnostic efficacy of serologic testing, symptoms, and the cycle threshold was not determined.

## Conclusion

PCR combined with the serologic testing algorithm greatly improved the yield and efficiency of the hunting of SARS-CoV-2 infection. The study provides a reference for a high-performance screening algorithm of PCR combined with serologic testing for the early detection of virus epidemics.

## Data availability statement

The original contributions presented in the study are included in the article/supplementary material, further inquiries can be directed to the corresponding author/s.

## Ethics statement

The studies involving human participants were reviewed and approved by (#xmzsyyky2021196) Institutional Ethics Committee of Zhongshan Hospital of Xiamen University, School of Medicine, Xiamen University. Written informed consent from the participants' legal guardian/next of kin was not required to participate in this study in accordance with the national legislation and the institutional requirements.

## Author contributions

X-ML had full access to all the data in the study, took responsibility for the integrity of the data and the accuracy of the data analysis, and supported funding. L-LF and X-ML contributed to the protocol and design of the manuscript. L-LF, J-HZ, and X-ML drafted the manuscript. All authors designed and conducted the statistical analysis, and accessed and verified the underlying data. All authors critically revised the manuscript for important intellectual content. All authors contributed to the article and approved the submitted version.
